# Proinflammatory cytokines modify Ca^2+^ signaling and induce an apoptotic lipidome in murine small intestinal organoids

**DOI:** 10.3389/fphys.2026.1738447

**Published:** 2026-02-06

**Authors:** Svenja Mareike Pauer, Alina Schindler, Parviz Ghezellou, Bernhard Spengler, Martin Diener, Jasmin Ballout

**Affiliations:** 1 Institute for Veterinary Physiology and Biochemistry, Justus Liebig University Giessen, Giessen, Germany; 2 Institute for Inorganic and Analytical Chemistry, Justus Liebig University Giessen, Giessen, Germany; 3 Institute of Molecular Systems Medicine, Goethe University, Frankfurt am Main, Germany; 4 Institute for Cardiovascular Physiology, Goethe University, Frankfurt am Main, Germany

**Keywords:** apoptosis, Ca^2+^ signaling, chloride secretion, intestinal organoids, lipidomics, mouse, proinflammatory cytokines

## Abstract

**Background:**

Inflammatory bowel diseases (IBD) are associated with elevated levels of proinflammatory cytokines exerting a detrimental effect on the intestinal epithelium. Since contradictory studies regarding time-dependent actions on epithelial secretion exist, intestinal organoids were treated with a mix of proinflammatory cytokines.

**Methods:**

Long-term (1–5 days) effects of a cytokine mix consisting of tumor necrosis factor α, interleukin-1β and interferon-γ on murine small intestinal epithelial organoids were investigated using live cell imaging, immunofluorescent staining, qPCR, scanning microprobe matrix-assisted laser desorption/ionization mass spectrometry imaging (AP-SMALDI MSI), and liquid-chromatography tandem mass spectrometry (UHPLC MS/MS).

**Results:**

Treatment with the cytokine mix resulted in an enlarged organoid lumen. Stimulation of Cl^–^ secretion seems to underly this swelling since it was inhibited by bumetanide, a blocker of Na^+^-K^+^-2Cl^–^ cotransporters. Ca^2+^ imaging experiments revealed that the cytokine mix time-dependently enhanced the response to carbachol, a Ca^2+^-dependent secretagogue, which would favor Ca^2+^-dependent Cl^−^ secretion. This was concomitant with an upregulated gene expression of inositol-1,4,5-trisphosphate receptor type 1 (IP_3_R1) and stromal interaction molecule 2 (STIM2), which is involved in store-operated Ca^2+^ entry. The cytokines induced apoptosis in organoids confirmed by an increase in caspase 3-immunopositive cells. Lipidomic analysis using UHPLC MS/MS revealed an upregulation of ceramides, sphingomyelins and ether-linked lipids within the epithelial cells. AP-SMALDI MSI showed an accumulation of ceramide-positive (apoptotic) cells within the organoid lumen.

**Conclusion:**

Proinflammatory cytokines induce fluid secretion and transiently upregulate Ca^2+^ signaling concomitant with the induction of an apoptotic lipidome in small intestinal organoids.

## Introduction

Inflammatory bowel diseases (IBD) like Crohn’s disease or ulcerative colitis are characterized by massive alterations in the intestinal epithelium caused by chronic inflammation. Patients exhibit symptoms like abdominal pain, watery or bloody diarrhea, weight loss or even systemic symptoms like anemia or arthritis (Yu and Rodriguez, 2017). The pathomechanism of IBD is not yet completely understood. An epithelial barrier defect, known as “leaky gut”, enables infiltration of usually harmless components of the microbiome into deeper layers of the gut wall, triggering an inflammatory reaction through the release of proinflammatory cytokines and other mediators, which further damage the intestinal epithelium resulting in a vicious circle (Wittkopf et al., 2014).

The consequences are a disrupted epithelial barrier and higher net secretion due to changes in the composition of the tight junctions and the transport function of the intestinal epithelium (Sandle and Rajendran, 2025). Such a leaky barrier can be explained by, e.g., an upregulation of pore-forming (Mankertz et al., 2009) and downregulation of tightening (Prasad et al., 2005) claudins. This is paralleled by alterations in ion transport across the colonic epithelium, predominantly an inhibition of Na^+^ and water absorption (Hawker et al., 1980). Regarding the secretion of Cl^−^, conflicting results have been reported. In specimens from patients with Crohn’s disease or ulcerative colitis, no net secretion of Cl^−^, but a parallel downregulation of both the absorptive (mucosal → serosal) as well as the secretory (serosal → mucosal) unidirectional flux of this anion has been measured (Hawker et al., 1980). On the other hand, TNFα or IL-1β stimulate the secretion of Cl^−^ across human colonic epithelium within several minutes (Schmitz et al., 1996; Bode et al., 1998). Divergent results have also been reported for Ca^2+^-dependent Cl^−^ secretion. Published data ranged from a downregulation of the secretory response induced by the Ca^2+^-dependent secretagogue carbachol in models of chemically induced experimental colitis (Pérez-Navarro et al., 2005) to a strong potentiation of Ca^2+^-dependent secretion in the human colonic tumor cell line, HT29 cl.19A, by TNFα (Oprins et al., 2000).

Another effect of proinflammatory cytokines, in particular TNFα, is the induction of apoptosis, leading to defects in the intestinal epithelium (Gitter et al., 2000). During programmed cell death, not only cellular proteins or nucleic acids are degraded, but there are also changes in cellular lipid composition (Boldyreva et al., 2021), e.g., the well-known translocation of phosphatidylserine from the inner to the outer leaflet of the plasma membrane where it serves as an “eat me” signal (Chaurio et al., 2009) for phagocytic cells in order to remove the apoptotic material.

Intestinal organoids offer a suitable model to investigate many functions of the intestinal epithelium due to their physiological composition of intestinal cell types. In contrast to primary epithelial cells with their limited viability, they allow to study also long-term effects of proinflammatory cytokines such as TNFα (Di Giorgio et al., 2023; Pauer et al., 2024; Sandle and Rajendran, 2025). In the present study, we used murine small intestinal organoids to investigate the effect of a mix of three proinflammatory cytokines relevant for IBD, i.e., TNFα, IL-1β and IFN-γ (Singh et al., 2016), which exert additive effects, e.g., on ion transport, when administered in combination (Schmitz et al., 1996; Bode et al., 1998).

With this study we focused on the following questions:

1. How does a mix of proinflammatory cytokines change epithelial functions regarding secretion and epithelial barrier?
2. Are there time-dependent effects in Ca^2+^ signaling?
3. Do cytokines, which are known to induce apoptosis, alter the lipidomic profile of the intestinal epithelium?

For this purpose, changes in transepithelial ion secretion, Ca^2+^ signaling, apoptosis and lipid profiles were investigated by morphometry, live cell imaging, immunofluorescent staining, qPCR, lipidomic analysis via ultra-high performance liquid-chromatography tandem mass spectrometry (UHPLC MS/MS), and atmospheric-pressure scanning microprobe matrix-assisted laser desorption/ionization mass spectrometry imaging (AP-SMALDI MSI). The combination of the latter two methods allows not only the untargeted analysis of changes in the lipidome by UHPLC MS/MS, but also the precise localization of the observed changes down to a lateral resolution of 1.4 µm via AP-SMALDI MSI (Kompauer et al., 2017).

## Materials and methods

### Animals

Intestinal stem cells for organoid cultures were isolated from small intestines pooled from two to four C57BL6/J mice of both sexes with the age of 5–9 days. Mice were killed by decapitation. Overall, three independent organoid cultures from nine animals were used for the present study. The mice were bred and housed at the central laboratory animal husbandry of the Justus Liebig University Giessen at a standard temperature of 23 °C–25 °C and air humidity of 50%–55% on a 12:12 h light-dark cycle with free access to water and food *ad libitum*. The experiments were approved by the named animal welfare officers of the Justus Liebig University Giessen (administrative number: 679_M and 879_M) and performed according to the German and European animal welfare law.

### Solutions

For the organoid culture, IntestiCult^™^ Organoid Growth Medium (Mouse; StemCell Technologies, Vancouver, Canada) was used. The buffer for Ca^2+^ imaging experiments consisted of 140 mmol/L NaCl, 5.4 mmol/L KCl, 10 mmol/L HEPES (N-(2hydroxyethyl)piperazine-N′-2-ethanesulfonic acid), 12.2 mmol/L glucose, 1.25 mmol/L CaCl_2_, and 1 mmol/L MgCl_2_. For Ca^2+^ imaging experiments with Ca^2+^-free buffer, CaCl_2_ was omitted. For the qPCR analysis as well as for the Ca^2+^ imaging experiments, organoids were removed from Matrigel® (#356231, growth-factor reduced, Corning, New York, USA) with 4 °C cold phosphate-buffered saline (ROTI®Cell PBS CELLPURE® #9143.1; ROTH, Karlsruhe, Deutschland) containing 0.1% (w/v) bovine serum albumin (PBS/BSA). For immunofluorescent stainings, a 100 mmol/L phosphate buffer (PB; containing 20 mmol/L NaH_2_PO_4_ and 80 mmol/L Na_2_HPO_4_) was used. pH of all buffers was set to 7.4 with 1 M NaOH/HCl.

### Intestinal organoid culture

For the cultivation of intestinal organoids, a modified protocol was used based on Sato et al. (2009) and the StemCell Technologies protocol (2016). For one organoid culture, small intestines were collected from two to four neonatal C57BL6/J mice. The jejunum was opened longitudinally, cut in 2 mm long segments and washed several times with ice-cold PBS until the supernatant was clear. The tissue was then transferred into ice-cold 2 mmol/L ethylenediaminetetraacetic acid (EDTA) dissolved in PBS (EDTA/PBS) and moved on a shaking plate (40 rpm) for 30 min to isolate crypts. If this resulted in a sufficient number of stem cell containing crypt fragments and single cells, the EDTA/PBS solution was removed under sterile conditions and exchanged with 10 mL ice-cold PBS/BSA. The cell suspension was pipetted up and down three times. After 30 s, the stem cell-containing suspension was transferred into a 50 mL tube through a 70 μm cell strainer (Cell Strainer, Corning®, New York, USA). The original pellet was resuspended with fresh PBS/BSA and the process was repeated three times. The fraction with the highest cell number and the lowest debris was selected, centrifuged (5 min at 280 g), resuspended with PBS/BSA, and transferred into a new 15 mL tube. After it was spun down again (5 min at 280 g), the supernatant was removed and the cell pellet was resuspended with an appropriate amount of IntestiCult^™^ Organoid Growth Medium Mouse (StemCell Technologies, Vancouver, Canada). The same volume of ice-cold Matrigel® was added by using pre-cooled pipette tips, carefully mixed and transferred to preheated (37 °C) well plates. When using 24-well plates (Corning® Costar®, # 3526), 50 µL drops of the Matrigel®/IntestiCult^™^ mixture were added to the center of each well; when using 48-well plates (Corning® Costar®, # 3548) only 25 µL were used. After solidification of the Matrigel® drops in the incubator (at 37 °C and 5% (v/v) CO_2_), 500 µL or 250 µL preheated IntestiCult^™^ was added to each well, respectively. Medium was changed every 2–3 days. For passaging of the organoids (every 5–7 days), the medium was removed and the Matrigel® was resolved using cold PBS/BSA. The organoids were disrupted mechanically by up and down pipetting. The solution was collected in a 15 mL tube, centrifuged for 5 min (280 g), supernatant was removed and the cell pellet was resuspended in the desired amount of IntestiCult^™^. Matrigel® was added and drops were formed as described above. To measure the effects of proinflammatory cytokines, the organoids were incubated with a mix of TNFα (100 ng/mL), IL-1β (20 ng/mL) and IFN-γ (100 pg/mL) for 1, 3 or 5 days. The administration of the cytokines started 1 day after the passaging for the study of long-term (5 days) effects, or 1–2 days after passaging for the study of short-term (1 and 3 days) effects. Cytokine mix was added again with every medium change.

### Swelling experiments

In order to record changes in the morphology of the organoids after exposure to the cytokine mix, time-lapse video recordings were performed over a period of 24 h. Photographs were taken every 5 min with an Olympus IX81 microscope equipped with a MT illumination system and an Olympus FireWire camera (V2.1.30). The microscope was mounted in a climatic chamber (Evotec AUC04/B) that kept the temperature at 37 °C with 5% (v/v) CO_2_ and air humidity at 50%. These live imaging experiments were performed with organoids in their Matrigel® dome covered with IntestiCult^™^ medium. The cytokine mix was added at the beginning of each experiment. After 22 h, forskolin (9 μmol/L), a cAMP-dependent secretagogue, was administered as viability control.

In order to investigate the sensitivity of the observed organoid’s swelling evoked by the cytokine mix to putative inhibitors, these were administered directly before the cytokine mix was applied. Photographs were taken of the organoids with the Nikon ECLIPSE Ts2R, HAMAMATSU ORCA-spark before and 24 h after incubation with the cytokine mix.

For the quantification of the observed swelling, the relative lumen size, expressed as luminal area in % of the total organoid’s area, was measured and calculated using ImageJ (1.53e, W. Rasband and contributors, National Institutes of Health, USA). For the 24 h time-lapse recordings, the relative lumen size was evaluated every hour, whereas for the blocking experiments using bumetanide and indometacin the change in relative lumen size before and 24 h after treatment was evaluated.

### Ca^2+^ imaging experiments

For Ca^2+^ imaging experiments, the same setup was used as for the swelling experiments. To prepare the organoids, Matrigel® was removed by gentle pipetting with ice-cold PBS/BSA, organoids were collected and centrifuged for 1 min at 200 g. The supernatant was discarded so that approximately 150 µL were left in the tube. After careful mixing, 30 µL of the cell suspension was transferred to a glass bottom microwell dish (MatTek Corporation, USA) covered with poly-L-lysin (0.1 mg/L, 70,000–150,000 kDa, Cell Systems, Troisdorf, Germany) and left for 10 min to let the organoids sink down. The organoids on the microwell dishes were loaded at room temperature with a solution containing the Ca^2+^-sensitive fluorescent dye Fura-2 acetoxymethylester (Fura-2/AM, 6 μmol/L) and an equal volume of the nonionic detergent pluronic acid (20% (w/v) stock solution in dimethyl sulfoxide (DMSO)) in CaCl_2_-containing buffer. After 60 min, the organoids were washed with 500 µL buffer, covered with 4 mL buffer (either with or without CaCl_2_) and transferred to the imaging setup. The emission ratio within a region of interest (ROI, set to the size of one individual cell) was calculated after alternately excitation at 340 and 380 nm. Data were sampled at 0.2 Hz. An increase in the Fura-2 ratio represents an increase in the cytosolic Ca^2+^ concentration.

When a stable baseline of the Fura-2 ratio was reached over 3 min, the acetylcholine derivative carbachol (50 μmol/L) was added to induce a Ca^2+^ influx into the cytosol of the epithelial cells. At the end of these experiments, 5 μmol/L cyclopiazonic acid (CPA), a blocker of the sarcoplasmic-endoplasmic reticulum Ca^2+^ ATPase (SERCA), i.e., the Ca^2+^ pump responsible for refilling the intracellular Ca^2+^-storing organelles (Moncoq et al., 2007), was administered as viability control.

For Ca^2+^ depletion/repletion experiments, Ca^2+^ stores were depleted in Ca^2+^-free buffer with thapsigargin (1 μmol/L), a SERCA inhibitor (Lytton et al., 1991). After about 12 min, CaCl_2_ (2.5 mmol/L) was resubstituted in the extracellular buffer to allow a capacitative Ca^2+^ influx into the epithelial cells.

The maximal increase in the rise of the Fura-2 ratio induced by administered drugs was measured as difference to the baseline (ΔFura-2 ratio). A response to the respective drugs were only accepted when two conditions were fulfilled simultaneously: 1.) the amplitude of the change had to exceed the 4-fold standard deviation of the scattering in the Fura-2 ratio during the basal time period just prior to drug administration. 2.) The amplitude of the change in the Fura-2 ratio exceeded an absolute value of 0.1. Non-viable cells, which did not respond to any drug with an increase in the Fura-2 ratio, were excluded from further analysis.

### qPCR

For qPCR experiments, the organoid-containing Matrigel® was dissolved with ice-cold PBS/BSA and the organoid suspension was centrifuged for 5 min at 280 g. The supernatant was discarded. The remaining cell pellet was resuspended with lysis buffer (Macherey-Nagel, Düren, Germany) and homogenized with a mixer mill (for 2 min at 30 Hz). The organoid samples were stored at −80 °C until RNA extraction was performed with the RNA Plus kit (Macherey-Nagel) according to manufacturers’ instructions. The concentration of RNA in each sample and their purity (OD260/280) was determined using the Nanodrop One^©^ (Thermo Fisher Scientific, Dreieich, Germany). To obtain an equal RNA concentration of 250 ng/mL, the samples were diluted with RNA/DNA-free water before transcribing into cDNA (High-Capacity RNA-to-cDNA Kit). To perform the qPCR experiments, TaqMan® Gene Expression Master Mix, the respective primers (see Table 1) as well as RNA/DNA-free water was added to the cDNA in accordance to manufactures’ instructions. In accordance with previous experiments with murine organoids exposed to TNFα, Gapdh and Gusb were chosen as reference genes (Pauer et al., 2024). If not indicated otherwise, primer and other qPCR supplies were obtained from Thermo Fisher Scientific.

**TABLE 1:** Primers used for qPCR.

Target gene	Assay ID	NCBI gene number
Gapdh	Mm99999915_g1	NM_008084.3
Gusb	Mm01197698_m1	NM_010368.1
CFTR	Mm00445197_m1	NM_021050.2
NKCC1 (SLC12A2)	Mm01265951_m1	NM_009194.3
TMEM16A (Ano1)	Mm00724407_m1	NM_001242349.1
IP_3_R1 (Itpr1)	Mm00439907_m1	NM_010585.5
IP_3_R2 (Itpr2)	Mm00444937_m1	NM_010586.2
IP_3_R3	Mm01306070_m1	NM_080553.3
SERCA1	Mm01275320_m1	NM_007504.2
SERCA2	Mm01201431_m1	NM_001110140.3
SERCA3	Mm00443898_m1	NM_001163336.1
Ryanodine receptor 1 (RyR1)	Mm01175211_m1	NM_009109.2
Ryanodine receptor 2 (RyR2)	Mm00465877_m1	NM_023868.2
Ryanodine receptor 3 (RyR3)	Mm01328421_m1	NM_177652.2
STIM1	Mm01158413_m1	NM_009287.4
STIM2	Mm01223103_m1	NM_001081103.2
Orai1	Mm00774349_m1	NM_175423.3
Orai2	Mm04214089_s1	NM_178751.3
Claudin 1 (Cldn-1)	Mm01342184_m1	NM_016674.4
Claudin 2 (Cldn-2)	Mm00516703_s1	NM_016675.4
Claudin 3 (Cldn-3)	Mm00515499_s1	NM_009902.4
Claudin 4 (Cldn-4)	Mm00515514_s1	NM_009903.2
Claudin 5 (Cldn-5)	Mm00727012_s1	NM_013805.4
Claudin 7 (Cldn-7)	Mm00516817_m1	NM_016887.6
Claudin 8 (Cldn-8)	Mm00516972_s1	NM_018778.3
Claudin 12 (Cldn-12)	Mm01316510_m1	NM_001193659.2
Claudin 18 (Cldn-18)	Mm00517321_m1	NM_001194921.1
M_1_ receptor (M_1_)	Mm00432509_s1	NM_001112697.1
M_2_ receptor (M_2_)	Mm01701855_s1	NM_203491.4
M_3_ receptor (M_3_)	Mm00446300_s1	NM_033269.4
G_q_α protein (Gnaq)	Mm00492381_m1	NM_008139.5
Phospholipase C β3 (Plcb3)	Mm00476953_m1	NM_001290349.1

For each primer, three technical and three biological replicates (i.e. 3 independent organoid passages) were measured per plate. Each qPCR protocol was run at least twice. To be able to compare the results, the individual efficiency for each primer was calculated with LinRegPCR (Version 2021.2; J.M. Ruijter, Amsterdam UMC, Netherlands). Samples, which did not reach a plateau before 40 cycles, as well as outliers (deviating more than ±5% of the mean efficiency for each primer) were excluded. The baseline corrected C_t_ values from each amplicon were calculated and used for comparison of expression levels with the efficiency-corrected ΔC_t_ method.

### Immunofluorescent staining

To identify apoptotic cells, immunofluorescent stainings of cleaved caspase 3 (Cas3)-positive cells were performed in combination with villin to identify the luminal border of the organoids. For this, the medium was removed from each well and the organoids (still in Matrigel®) were fixed with 4% (w/v) paraformaldehyde (PFA; 37 °C) for 30 min at room temperature. Afterwards, the organoids were washed twice with warm PBS/BSA (to avoid a further dissolution of the Matrigel®) before collecting the organoids in a 15 mL tube using 4 °C cold PBS/BSA to dissolve the Matrigel®. After a settling time of 10 min, the supernatant was removed until about 1 mL suspension remained. This volume was transferred into a 1.5 mL tube, spun down for 1 s (microlitre centrifuge; Roth, Karlsruhe, Germany) before the supernatant was discarded. The remaining cell pellet was resuspended with liquid gelatin (15%, w/v, 37 °C) and poured into a small mold that was mounted to a motor which rotated it at −20 °C during cool down of the gelatin. After 30 min, the gelatin block was removed and stored at −80 °C overnight. For immunofluorescent stainings, 4 µm thick slices were cut with a cryostat (Leica CM3050 S, Leica, Wetzlar, Germany) and transferred to microscope slides (Superfrost® Plus, Thermo Fisher Scientific). The slides were rehydrated with PB before they were incubated for 2 h with a blocking solution containing PB with 0.2% (v/v) Triton-X-100, 3% (w/v) BSA, and 10% (v/v) donkey serum. The antibodies against Cas3 and Villin (Table 2) were added to an antibody solution containing 0.1% (v/v) Triton-X-100, 1% (w/v) BSA, 0.5% (w/v) milk powder, and 1% (v/v) donkey serum in PB. The negative controls were treated only with this solution without the respective antibodies. The sections were incubated overnight at 4 °C before washing with PB (3x for 5 min) and incubating with the secondary antibody (Table 2) at room temperature for 1 h. After washing with PB (3x for 5 min), the samples were embedded in RotiFluo with DAPI (4,6-diamidio-2-phenylindoldilactate; Roth) for nucleus staining. The pictures were taken with a fluorescence microscope (Nikon 80i; Nikon, Düsseldorf, Germany). Only those cells, which showed a positive signal for DAPI and Cas3 were counted in a blinded fashion for quantitative analysis.

**TABLE 2:** Antibodies used for immunofluorescent staining.

Target	Host	Supplier, catalog (#) and lot number	Research resource identifier (RRID) number	Dilution
*Primary antibodies*				
Cleaved caspase 3	Rabbit	Cell signaling Technology (#9661)	RRID:AB_2341188	1:500
Villin	Mouse	Santa cruz (#sc-58897; Lot: E0119)	RRID:AB_2304475	1:100
*Secondary antibodies*				
Cy3 rabbit IgG	Donkey	Jackson ImmunoResearch (#711–165-152; Lot: 140443)	RRID:AB_2307443	1:1000
Alexa 488 mouse IgG	Donkey	Thermo Fisher scientific (#A21202; Lot: 2563848)	RRID:AB_141607	1:250

### AP-SMALDI MSI

AP-SMALDI MSI (atmospheric-pressure scanning microprobe matrix-assisted laser desorption/ionization mass spectrometry imaging) is a special form of MALDI, performed under atmospheric pressure and optimized for highest lateral resolution. MALDI-based mass spectrometry imaging proved to be a reliable technique to visualize the localization of the observed changes in the lipidome within the organoids. While conventional imaging techniques are able to visualize only a few targeted compounds in parallel, mass spectrometry imaging instead provides a straightforward and untargeted generation of spatial distribution images of all detectable biomolecules. The most widely used ionization method for mass spectrometry imaging is matrix-assisted laser desorption/ionization (MALDI), a “soft ionization method” enabling the investigation of larger molecules, such as lipids. In this method, a pulsed laser beam is employed to ablate material from a sample embedded in a matrix. The laser moves across the sample in a rasterizing manner to generate a mass spectrum for each spot. Ion images can then be generated for each detected signal, having distinct mass-to-charge-number (*m*/*z*) ratios, thus representing the spatial distribution of the compound belonging to the signal. While the *m*/*z* ratio can be used to determine the sum formula of the corresponding compound, it cannot be used to assign the chain lengths in lipids or to determine the position of double bonds (Römpp and Spengler, 2013).

For AP-SMALDI MSI experiments, the organoids were fixed using 4% (w/v) PFA in PB and embedded in 15% (w/v) pure gelatin (Sigma, Taufkirchen, Germany) from porcine skin dissolved in distilled water before they were frozen by −80 °C. The organoid-containing gelatin blocks were then cut into 20 µm thick sections using a cryotome (HM525 cryostat; Thermo Fisher Scientific, Bremen, Germany) and placed on glass slides. Sections were stored at −80 °C until further use. Microscopic light images of the cryosections were recorded using a digital light microscope (VHX-5000, KEYENCE, Neu-Isenburg, Germany) with 500-fold magnification.

Prior to matrix application, the sections were thawed in a desiccator for 30 min to prevent water precipitation. A high-resolution matrix preparation system (SMALDIPrep; TransMIT GmbH, Giessen, Germany) was used to ensure uniform deposition of matrix solutions. For positive-ion mode measurements, 70 µL of freshly prepared 2,5-dihydroxybenzoic acid (DHB) solution (30 mg/mL in 50:50 acetone/water with 0.1% TFA) was applied. For negative-ion mode measurements, 120 µL of 9-aminoacridine solution (7 mg/mL in 70:30 ethanol/water) was deposited. AP-SMALDI MSI experiments with a step size of 3 µm were performed with an AP-SMALDI^5^ AF ion source (TransMIT GmbH, Giessen, Germany) coupled to an orbital trapping mass spectrometer (Thermo Scientific Q Exactive HF, Thermo Fisher Scientific (Bremen) GmbH, Bremen, Germany) with a mass resolution of 240,000 at *m/z* 200. All corresponding parameters are listed in Supplementary Protocol S1.

The obtained data were converted into an imzML format using the “Raw-To-imzML” Converter. The Mirion software (TransMIT GmbH, Giessen, Germany; v3.3.64.22) was used for ion-image generation. The images were manually examined for distributions of relevant lipids, and corresponding red-green-blue (RGB) overlay images were created.

### UHPLC MS/MS

The lipidome of the organoids was extracted via a methyl tert-butyl ether (MTBE) extraction procedure using a LIPIDOMIX standard for semi-quantification. 300 μL cold methanol was added to 50 mg sample and homogenized with ceramic beads in a micro mill (Pulverisette 23, FRITSCH GmbH, Idar-Oberstein, Germany). The mixture was vortexed for 1 min at 950 rpm in a ThermoMixer C (Eppendorf SE, Hamburg, Germany) and incubated for 10 min on ice. Afterwards, 1 mL MTBE was added to the solution and vortexed at 2 °C and 950 rpm for 1 h. Then 250 µL H_2_O were added, vortexed for 10 min at 950 rpm and centrifuged (Centrifuge 5,804, Eppendorf SE, Hamburg, Germany) for 10 min at 10,000 rcf. The upper organic layer, which should contain the lipids, was separated and the lipid extraction was repeated for the residue. The organic layers were combined and vaporized using a nitrogen flow. The dried lipids were stored at −80 °C until further use.

The extracted lipids were separated using a reversed-phase 2.6 µm C18 column (100 mm × 2.1 mm; Kinetex, Phenomenex, Torrance, CA, USA) installed in an ultra-high performance liquid-chromatography (UHPLC) system (Ultimate 3000 UHPLC, Thermo Fisher Scientific (Bremen) GmbH, Bremen, Germany). The column was maintained at 40 °C and the analytes were separated in a stepwise gradient elution. Mobile phase A was a mixture of acetonitrile (ACN)/H_2_O (60:40) and mobile phase B of propan-2-ol (IPA)/ACN/H_2_O (90:8:2). Both mobile phases contained 0.1% (v/v) formic acid and 10 mmol/L ammonium formate. The injection volume was 50 µL with a constant flow rate of 0.25 mL/min. The gradient elution started at 20% mobile phase B, rising to 30% B over 4 min, to 45% over 2 min, to 60% B over 4 min and to 65% over 4 min 65% mobile phase B was held for another 4 min before rising the gradient to 90% B, and held for 2 min. Finally, the column was re-equilibrated with 20% B for 5 min.

Coupled to the UHPLC, a heated electrospray-ionization source (HESI) connected to an orbital trapping mass spectrometer (Q Exactive HF-X, Thermo Fisher Scientific (Bremen) GmbH, Bremen, Germany) recorded the corresponding tandem (MS/MS) mass spectra in positive- and negative-ion mode. All source parameters are listed in Supplementary Table S2.

The obtained data were converted into an ABF-file using Reifycs Analysis Base File Converter. MS-Dial LipidBlast (version 68) was used for lipid identification and annotation via MS-Dial (v5.1.230912). All MS-Dial parameters are listed in Supplementary Table S3. The results were normalized based on the LIPIDOMIX standard and filtered for MS^2^-acquired and reference-matched lipids. The final data were exported into a normalized-peak-area table for subsequent statistical analysis. Statistical analysis was performed using MetaboAnalyst 6.0. A low-repeatability filter was used with a relative standard deviation greater than 25% and a low-variance filter of 40%. A False Discovery Rate (FDR)-adjusted p-value of 5% (q-value 0.05) was used for correction across all test results. The data were normalized by median and log-transformed. For the comparison of two groups, a Student’s t-test was performed.

### Chemicals

All chemicals used for UHPLC MS/MS and AP-SMALDI MSI experiments were at least of analytical grade. Ammonium formate was purchased from Thermo Scientific Chemicals (Thermo Fisher Scientific Inc., Waltham, MA, USA), acetone and trifluoroacetic acid (TFA) from Merck KGaA (Darmstadt, Germany), and SPLASH LIPIDOMIX from Avanti Research (Alabaster, AL, USA). 2,5-Dihydroxybenzoic acid (DHB), 4-hydroxy-3-methoxycinnamaldehyde (CA), formic acid (FA) and 2-methoxy-2-methylpropane (MTBE) were obtained from Sigma Aldrich (St. Louis, MO, USA). Acetonitrile (ACN), methanol (MeOH), propan-2-ol (IPA), and water were purchased from Avantor, Inc. (Radnor, PA, USA), 9-aminoacridine (9-AA) was from TCI (Eschborn, Germany).

### Drugs

Carbachol and LaCl_3_ were dissolved in distilled water, while bumetanide, forskolin, indometacin, and thapsigargin were dissolved in ethanol (final maximal ethanol concentration 0.5% (v/v)). Dimethyl sulfoxide (DMSO) was used as solvent for cyclopiazonic acid (CPA; maximal DMSO concentration 0.1% (v/v)). Recombinant TNFα as well as IL-1β (Thermo Fisher Scientific gibco^™^ # PMC0811) were dissolved in sterile PBS/BSA (0.1%). IFNγ (Thermo Fisher Scientific gibco^™^ # PMC4031) was diluted in sterile PBS/BSA. If not labeled differently the drugs were obtained from Sigma, Taufkirchen, Germany.

### Statistics and data handling

In general, results are given as mean ± standard error of the mean (SEM) with number (n) of investigated organoids or cells. Relative expression ratios in the qPCR experiments are given as arithmetic mean ±95% confidence interval. Experiments were repeated at least 3 times with three independent organoid passages. For comparison of two groups, Student’s t-test or Mann-Whitney U-test was performed. In order to find out which test method has to be used, an F-test was applied. For comparison of more than two groups, analysis of variance (ANOVA) was used followed by Tukey’s *post hoc* test.

In order to compare the enhancement of a biological response, i.e., the enhancement (ΔΔFura) of the carbachol-induced rise (ΔFura) in the Fura-2 signal by cytokines in the absence and presence of La^3+^, linear contrasts were analyzed with:

$$\begin{array}{ll} \Delta \Delta \text{Fura} & =\left(\Delta {\text{Fura}}_{+\text{cytokine}}\;–\;\Delta {\text{Fura}}_{-\text{cytokine}}\right)\\ & \;-\left(\Delta {\text{Fura}}_{+\text{cytokine}+\text{La}}\;–\;\Delta {\text{Fura}}_{-\text{cytokine}+\text{La}}\right) \end{array}$$

Statistical significance was calculated by linear contrast analysis using the Scheffé test. The SEM of the resulting difference was obtained using the law of Gaussian error propagation. Statistical analysis of C_t_ values was performed with REST^©^ (REST 2009) for group-wise comparison of qPCR data (Pfaffl et al., 2002). P < 0.05 was considered to be statistically significant.

## Results

### Morphological changes induced by cytokine exposure

**FIGURE 1:**
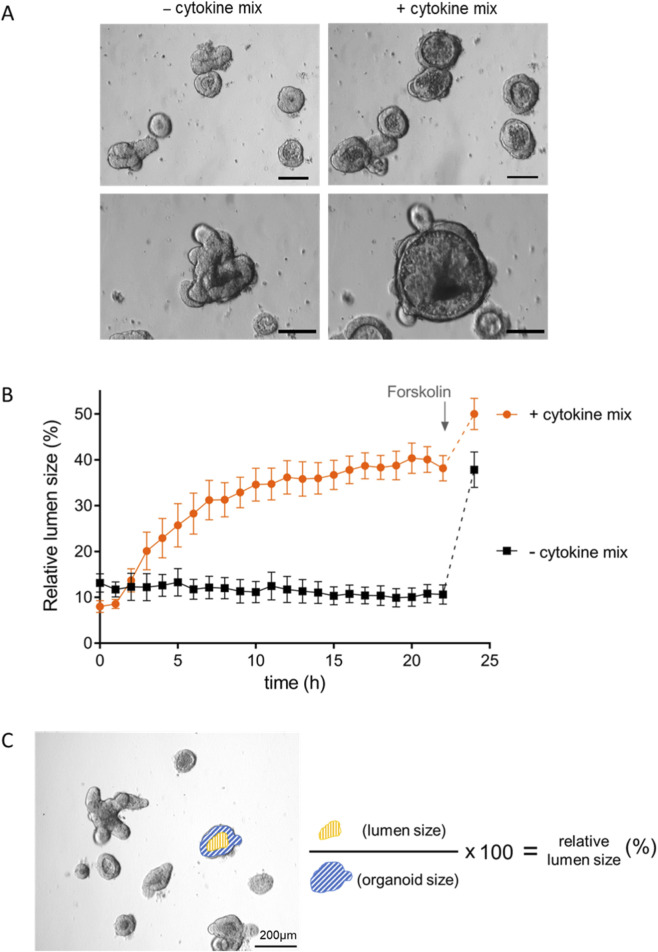
**(A)** Morphological changes induced by incubation with the cytokine mix. Bright-field microscopical images of organoids before (left) and 24 h after incubation with the cytokine mix (TNFα (100 ng/mL) + IL-1β (20 ng/mL) + IFN-γ (100 pg/mL)) (right) at two different magnifications (enlarged in the lower row). The scale bar in each picture equals 100 µm. Pictures were taken with the Nikon ECLIPSE Ts2R, HAMAMATSU ORCA-spark, 10x lens, NIS Elements 2.30 software (Nikon). **(B)** Time course of the swelling induced by the proinflammatory cytokines. Changes in relative lumen size over a period of 22 h in untreated organoids (black) and organoids incubated with the cytokine mix (orange) at the beginning of the experiment. Forskolin (9 μmol/L) was administered as viability control after 22 h. Data are the mean of relative lumen size in % ± SEM of n = 14–15 organoids per group from three independent passages per group. **(C)** shows an example of how the relative lumen size was measured and calculated (size of the lumen/size of the entire organoid ·100).

Under control conditions, the organoids exhibited typical crypt and villus domains resembling the organization of the intestinal epithelium within the mucosa of the small intestine (Figure 1A). This morphology changed when the organoids were treated with a cytokine mix containing IL-1β (20 ng/mL), IFN-γ (100 pg/mL) and TNFα (100 ng/mL). The organoids appeared to round off, and the differentiation between crypt and villus domains got less pronounced. This swelling was concomitant with an apparent flattening of the epithelium and an increased accumulation of cells and debris in the lumen (Figure 1A). Analysis of the circularity with ImageJ (defined as (4π*area)/perimeter^2^; 1 = perfect circle; 0 = elongated polygon) revealed a trend for a more roundedness after cytokine incubation (0.78 ± 0.03, n = 15 organoids) compared with untreated controls (0.66 ± 0.05, n = 14 organoids), albeit this did not reach statistical significance (p = 0.067).

### Time course of the cytokine mix-induced swelling

To investigate the time course of these morphological changes induced by the cytokine mix, time-lapse experiments over a period of 24 h were performed and changes in the relative lumen size, i.e., the luminal area in % of the total organoid’s area, were quantified (Figure 1C). When the cytokine mix was administered, the organoids remained unaltered for about 1 h. After this delay, an increase in relative lumen size started with a half-time of 6.4 ± 0.95 h, which finally amounted to 40% of the total organoid area (Figure 1B). Within 12 h, the swelling was nearly complete as it had reached about 90% of the maximal induced swelling response (Figure 1B). Organoids which were not treated with the cytokine mix served as time-dependent controls. Their relative lumen size, ranging from 10% to 15% of the total area covered by an individual organoid, remained stable over a period of 22 h (Figure 1B). After 22 h, the secretagogue forskolin (9 μmol/L), which stimulates adenylate cyclases (Seamon and Daly, 1981) and thereby induces a cAMP-dependent Cl^−^ secretion, was administered as viability control. In untreated controls the forskolin-induced changes in relative lumen size reached 38%, whereas it increased to 50% after cytokine mix incubation (Figure 1B).

### Cl^−^ secretion is involved in cytokine mix-induced swelling

**FIGURE 2:**
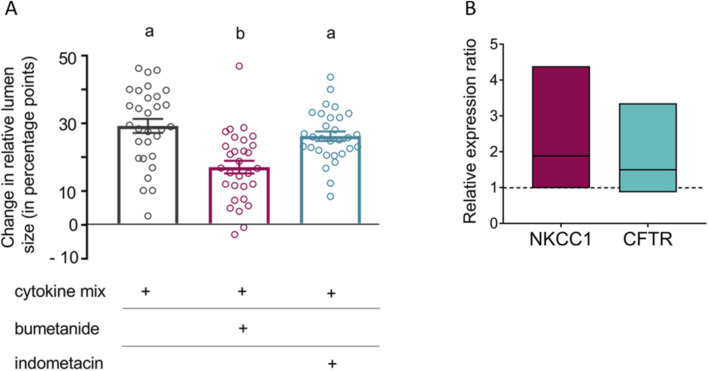
**(A)** Changes in relative lumen size (in percentage points) after 1 d incubation with the cytokine mix compared to organoids treated with cytokine mix + bumetanide (100 μmol/L) and organoids treated with cytokine mix + indometacin (100 μmol/L). Data are given as difference between relative lumen size at the end (24 h) and the beginning of the experiment and are presented as mean (bars) ± SEM (lines) with single values as symbols, n = 30 organoids per group originating from at least three independent passages. Statistically homogeneous groups are marked with the same letter (a, b; p < 0.05; ANOVA followed by Tukey’s *post hoc* test). **(B)** Relative expression of NKCC1 and CFTR in organoids treated with the cytokine mix (TNFα (100 ng/mL) + IL-1β (20 ng/mL) + IFN-γ (100 pg/mL)) compared to untreated controls (dotted line at 1). Results were normalized to the reference genes Gapdh and Gusb. Data are shown as arithmetic mean (horizontal line) ± 95% confidence interval, n = 3 biological replicates originating from independent passages.

In the case of TNFα, the cytokine-induced swelling was inhibited by bumetanide (Pauer et al., 2024). Bumetanide blocks Na^+^-K^+^-2Cl^–^ cotransporters (NKCC; Kaplan et al., 1996) such as the NKCC1, which is responsible for the basolateral uptake of Cl^–^ into the cell during intestinal Cl^−^ secretion (Haas and Forbush, 2000; Keely and Barrett, 2022). Thus, we investigated if this might also be the case for the organoid swelling induced by the cytokine mix. In the absence of bumetanide, the cytokine mix induced an increase in relative lumen size by 29.2 ± 2.1 percentage points. In contrast, in organoids treated with bumetanide (100 μmol/L) the cytokine mix-induced swelling amounted only 17.2 ± 1.9 percentage points, i.e., the NKCC inhibitor reduced it by about 40% (p < 0.05, Figure 2A). As proinflammatory cytokines can induce Cl^−^ secretion via the release of prostaglandins (Schmitz et al., 1996) which are produced by cyclooxygenases, indometacin (100 μmol/L), an inhibitor for these enzymes (Vane et al., 1998), was tested. However, indometacin had no effect on the swelling induced by the cytokine mix (Figure 2A).

To find out whether the cytokines might induce Cl^−^ secretion via upregulation of key transporters involved in transepithelial Cl^−^ transport such as the basolateral NKCC1 or the apical cystic fibrosis transmembrane regulator (CFTR) anion channel, qPCR experiments were performed. These experiments revealed only a numerical increase in the expression of both transporters measured after 3 days cytokine exposure, but did not reach statistical significance due to high scattering (Figure 2B).

### Cytokines modulate the Ca^2+^ response induced by the cholinergic agonist carbachol

Beside cAMP, another important second messenger involved in the induction of intestinal secretion is Ca^2+^ (see also Keely and Barrett, 2022). Due to the conflicting results reported in the literature concerning a possible down- or upregulation of Ca^2+^-dependent intestinal secretion under inflammatory conditions (see Introduction), we investigated in Ca^2+^ imaging experiments whether the cytokine mix might alter Ca^2+^ signaling when organoids were stimulated with carbachol to induce the Ca^2+^-dependent Cl^−^ secretion.

**FIGURE 3:**
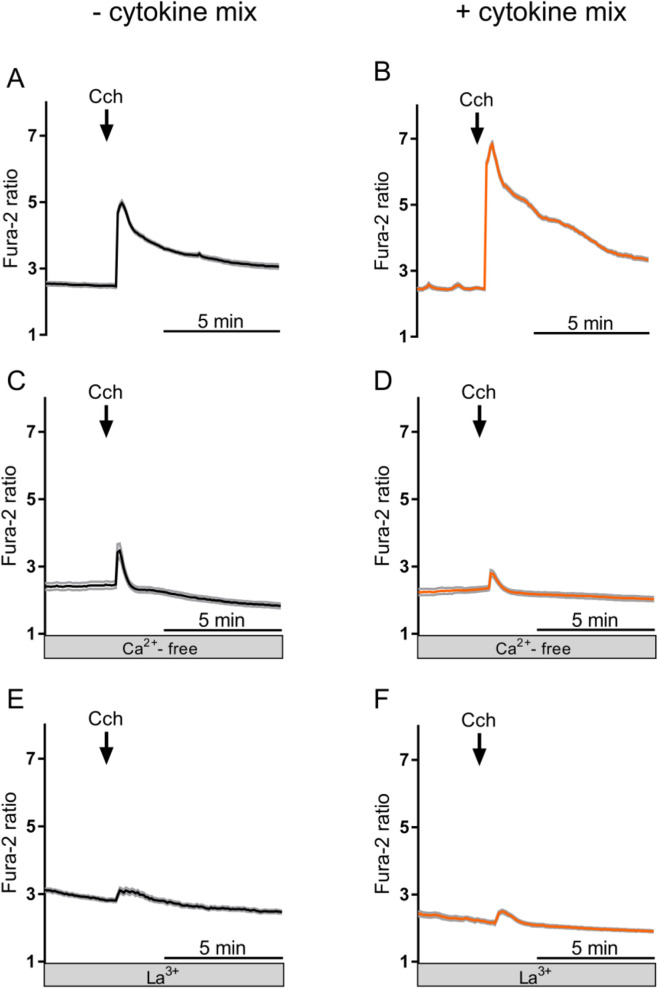
Representative curves of changes in the Fura-2 ratio of untreated **(A,C,E)** and cytokine mix-treated organoids (for 1 d; **(B,D,F)**) after stimulation with carbachol (Cch; 50 μmol/L) in Ca^2+^-containing buffer **(A,B)** or Ca^2+^-free buffer **(C,D)**. **(E)** and **(F)** show the response to carbachol after treatment with lanthanum ions (La^3+^; 1 mmol/L LaCl_3_) in Ca^2+^-containing buffer. Data are means (thick lines) ± SEM (gray lines), n = 9–16 cells in each organoid; for statistics, see Tables 3 and 4.

In Ca^2+^-containing buffer, carbachol evoked a biphasic increase in the Fura-2 ratio consisting of a fast, transient peak followed by slowly declining plateau phase (Figure 3A). These two phases are known to be caused by an initial release of Ca^2+^ from intracellular stores followed by a sustained influx from the extracellular space via capacitative Ca^2+^ entry (Lindqvist et al., 1998). Under control conditions, i.e., without cytokine treatment, the maximal increase in the Fura-2 signal amounted to 1.62 ± 0.09. This response was significantly enhanced by about 50% after 1 d cytokine exposure (Figure 3B; Table 3). This difference between the cytokine-treated and untreated organoids got smaller and lost statistical significance with prolonged (≥3 days) cytokine exposure (Table 3).

**TABLE 3:** Ca^2+^ imaging of organoids (± cytokine mix) in Ca^2+^-containing and Ca^2+^-free buffer.

Buffer (± Ca^2+^)	Treatment		Basal Fura-2 ratio		Carbachol-induced ΔFura-2 ratio		Cells responding to carbachol	
							%	n
Ca^2+^-containing buffer	1d	‒ cytokine mix	3.14 ± 0.07		1.62 ± 0.09		100	62/62
		+ cytokine mix	3.23 ± 0.07		2.46 ± 0.17*	↑	100	60/60
	3d	‒ cytokine mix	3.36 ± 0.07		1.01 ± 0.09		86	51/59
		+ cytokine mix	2.32 ± 0.06*	↓	1.25 ± 0.14		98	64/65
	5d	‒ cytokine mix	3.12 ± 0.09		1.57 ± 0.10		96	55/57
		+ cytokine mix	2.25 ± 0.05*	↓	1.73 ± 0.08		100	61/61
Ca^2+^-free buffer	1 d	‒ cytokine mix	2.68 ± 0.06^#^		0.81 ± 0.05^#^		76	53/70
		+ cytokine mix	2.56 ± 0.06^#^		0.48 ± 0.03^#^*	↓	95	62/65
	3 d	‒ cytokine -mix	3.14 ± 0.06^#^		0.85 ± 0.06		100	64/64
		+ cytokine mix	2.51 ± 0.07^#^*	↓	1.49 ± 0.08^#^*	↑	100	67/67
	5 d	‒ cytokine mix	2.84 ± 0.05^#^		0.91 ± 0.07^#^		98	65/66
		+ cytokine mix	2.06 ± 0.07*	↓	1.31 ± 0.11^#^*	↑	100	61/61

The effect of carbachol (50 μmol/L) on the Fura-2, ratio (reflecting the cytosolic Ca^2+^ concentration) of intestinal organoids with or without pretreatment with cytokine mix (TNFα (100 ng/mL) + IL-1β (20 ng/mL) + IFNγ (100 pg/mL)) is shown as increase in Fura-2, ratio (ΔFura-2, ratio) compared to baseline levels. Data are means ± SEM, n is given in % and as absolute number of cells responding to carbachol in comparison to the total number of viable cells investigated (for viability criteria, see Materials and methods). Organoids from at least three independent passages were used. *p < 0.05 compared to the corresponding parameter without cytokine mix. ^#^p < 0.05 compared to the corresponding parameter in Ca^2+^-containing buffer. ↓ or ↑ symbolize the manner of change.

The effect of cytokine exposure on the carbachol-induced Ca^2+^ signal was drastically changed, when the experiments were carried out under Ca^2+^-free conditions. In the absence of extracellular Ca^2+^, carbachol induced only a monophasic rise in the cytosolic Ca^2+^ concentration reflecting a Ca^2+^ release from intracellular stores (Figure 3C). In contrast to its stimulatory effect in Ca^2+^-containing buffer, this response was reduced after 1 d of cytokine mix treatment (Figure 3D; Table 3), but enhanced after longer (≥3 days) cytokine exposure (Table 3). This suggests that besides the prominent effect of cytokines on Ca^2+^ influx from the extracellular space, they also modulate–in an opposite manner–Ca^2+^ release from Ca^2+^-storing organelles.

### Stimulation of capacitative Ca^2+^ entry by short-term cytokine exposure

**FIGURE 4:**
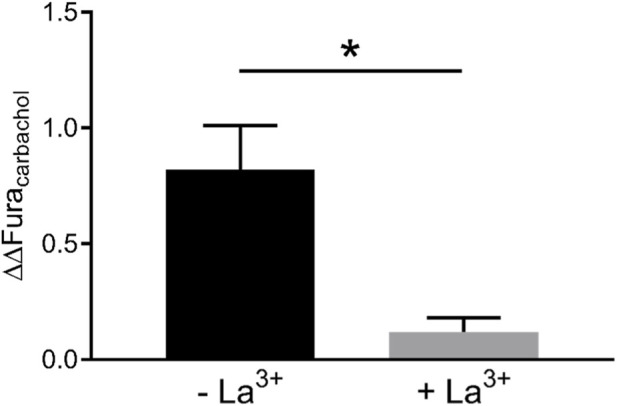
Enhancement of the response to carbachol (50 μmol/L) after treatment with the cytokine mix for 1 d in the presence (+La^3+^) and absence (– La^3+^) of lanthanum ions (1 mmol/L LaCl_3_). The enhancement of the carbachol response by cytokine treatment (ΔΔFura_carbachol_) was calculated from the data presented in Table 3 with the following formula: ΔΔFura_carbachol_ = (ΔFura_+cytokine_–ΔFura_-cytokine_) – (ΔFura_+cytokine+La_–ΔFura_-cytokine+La_). Data are presented as means +SEM (for number of experiments, see Tables 3 and 4). Statistically significant differences (*p < 0.05) were calculated using a linear contrast analysis by a Scheffé test.

Two further control experiments were performed to prove that short-term (≤1 d) cytokine exposure enhances Ca^2+^ influx from the extracellular space. In the first experimental series, the organoids were kept in a buffer containing lanthanum chloride (LaCl_3_), i.e., a blocker of nonselective cation channels which are involved in the capacitative Ca^2+^ entry in the intestinal epithelium (Frings et al., 1999). Lanthanum ions (La^3+^) strongly inhibited the rise in the Fura-2 signal (ΔFura-2) induced by carbachol (cf. Figures 3A,E,B,F). In those experiments, where organoids were pretreated with La^3+^, the stimulatory effect of cytokine mix pretreatment on the carbachol-induced ΔFura-2 was reduced by more than 85% (Figure 4).

As further approach, Ca^2+^ depletion/repletion experiments were performed. For this purpose, Ca^2+^ stores were emptied by treating the organoids with thapsigargin, an inhibitor of sarcoplasmatic/endoplasmatic reticulum Ca^2+^ ATPases (SERCA; Lytton et al., 1991), in the absence of extracellular Ca^2+^. When Ca^2+^ is then replenished in the extracellular medium, it will flow into the cell inducing an increase in the cytosolic Ca^2+^ concentration. Without cytokine mix pretreatment, Ca^2+^ repletion induced a rise in the Fura-2 signal of 1.38 ± 0.07 in 87 from 89 cells tested. After 1 d of cytokine exposure, this response was numerically larger as it amounted to 1.61 ± 0.10 (n = 96 from 96 cells tested). Although this difference did not reach statistical significance, the numeric increase fits well to the assumption that shorter (≤1 d) cytokine exposure stimulates Ca^2+^ entry from the extracellular space into the epithelial cells.

### Changes in the expression of transporters and signaling molecules involved in Ca^2+^ signaling

**FIGURE 5:**
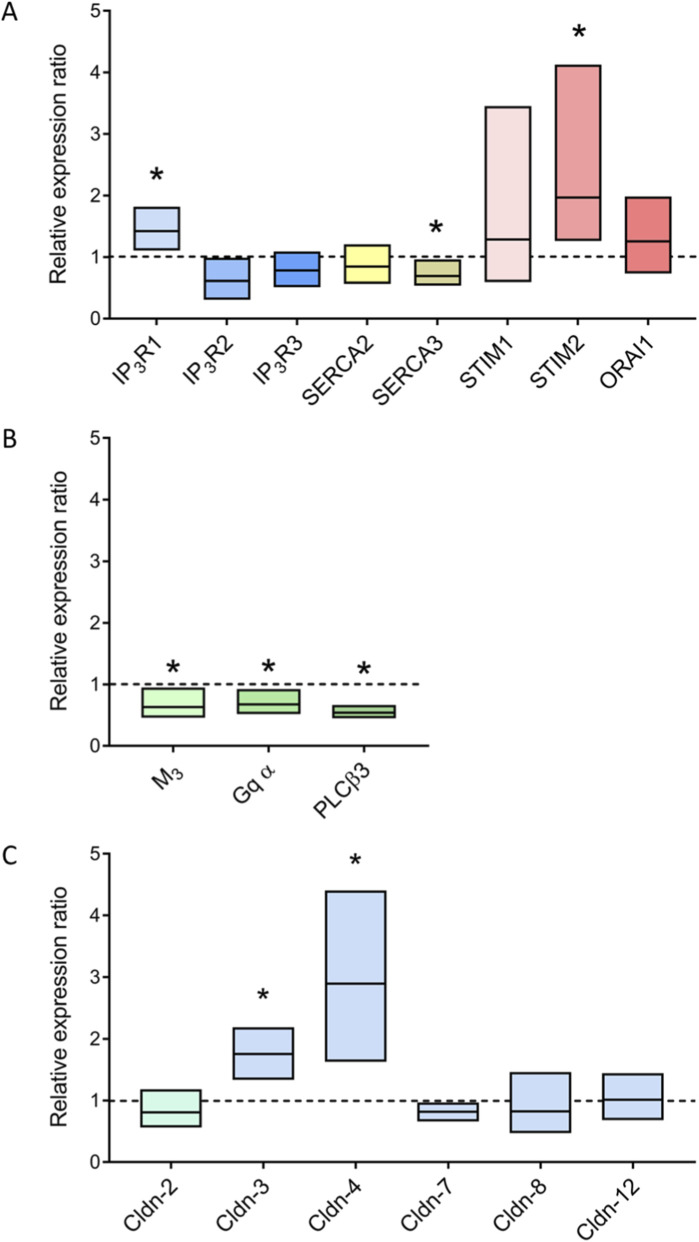
Relative gene expression of proteins involved in Ca^2+^ signaling **(A)**, cholinergic signaling **(B)** or of pore-forming (mint) and tightening (blue) claudins **(C)**. Organoids treated with the cytokine mix for 3 days were compared to untreated control organoids (dotted line at 1). Results were normalized to the reference genes Gapdh and Gusb. Data are shown as arithmetic mean (horizontal line) ± 95% confidence interval, n = 3 biological replicates originating from independent passages.

In order to find out whether the cytokine mix modifies the expression of transporters or signaling molecules involved in Ca^2+^-dependent Cl^−^ secretion, qPCR experiments were performed. A cytokine mix exposure of 3 days was selected to obtain a sufficient time span for putative regulation on the transcriptional level. IP_3_ receptor type 1 (IP_3_R1) and stromal interaction molecule (STIM) 2, a protein responsible for measuring the Ca^2+^ level inside the endoplasmic reticulum (Prakriya and Lewis, 2015), were significantly upregulated in organoids after pretreatment with the cytokine mix (Figure 5A). Together with a numeric trend for an enhanced expression of Orai1 (Figure 5A), this might fit well to the observed increase in Ca^2+^ influx from the extracellular space via capacitative Ca^2+^ entry (Figures 3, 4; Tables 3 and 4). SERCA3 was slightly downregulated by the cytokine mix (Figure 5A), which would also lead to a stronger store depletion and thereby an enhanced capacitative Ca^2+^ entry. No consistent amplifications of the mRNA for the different subtypes of ryanodine receptors (RyR1 - RyR3), SERCA1, Orai2 or TMEM16A were found.

**TABLE 4:** Ca^2+^ imaging with lanthanum pre-treatment.

Buffer (± Ca^2+^)	Treatment	Carbachol-induced ΔFura-2 ratio		Cells responding to carbachol	
				%	n
Ca^2+^-containing buffer	‒ cytokine mix	0.37 ± 0.04		74	45/61
	+ cytokine mix (1d)	0.49 ± 0.04*	↑	77	50/65

Carbachol-induced increase in the Fura-2, ratio (Δ Fura-2, ratio) after blocking of cation channels with lanthanum ions (La^3+^) in untreated organoids and organoids treated with the cytokine mix for 1 d. Data are means ± SEM, n is given in % and as absolute number of cells responding to carbachol in comparison to the total number of viable cells investigated. Organoids from at least three independent passages were used. *p < 0.05 compared to the response without cytokine mix treatment. ↓ or ↑ symbolize the manner of change.

Interestingly, the mRNA for signaling molecules involved upstream Ca^2+^ release/Ca^2+^ influx, i.e., the muscarinic receptors type M_3_, the G-protein subunit G_q_α as well as the phospholipase Cβ3 (PLCβ3) were significantly downregulated when organoids were incubated with the cytokine mix compared with the untreated controls (Figure 5B).

### Gene expression of epithelial barrier proteins

During intestinal inflammation, the integrity of the epithelial barrier is an important pathophysiological factor since an intact barrier is a prerequisite to prevent the entry of microbiota and putative toxins (Sandle and Rajendran, 2025). The paracellular permeability of the epithelium is mostly determined by tight junction components, especially claudins, of which some are pore forming while others are known to tighten the epithelial barrier (Garcia-Hernandez et al., 2017). In order to find out, whether the cytokine mix affects the expression of relevant claudins in the organoid model, qPCR experiments for claudins 1, 2, 3, 4, 5, 7, 8, 12 and 18 were performed. The expression of claudins 2, 7, 8 and 12 did not differ between both groups while claudins 1, 5 and 18 were not detected. Surprisingly, there was a significant upregulation of tightening claudins 3 and 4 after incubation with the cytokine mix compared with untreated control organoids (Figure 5C).

### Cas3-immunopositive cells as apoptosis marker

**FIGURE 6:**
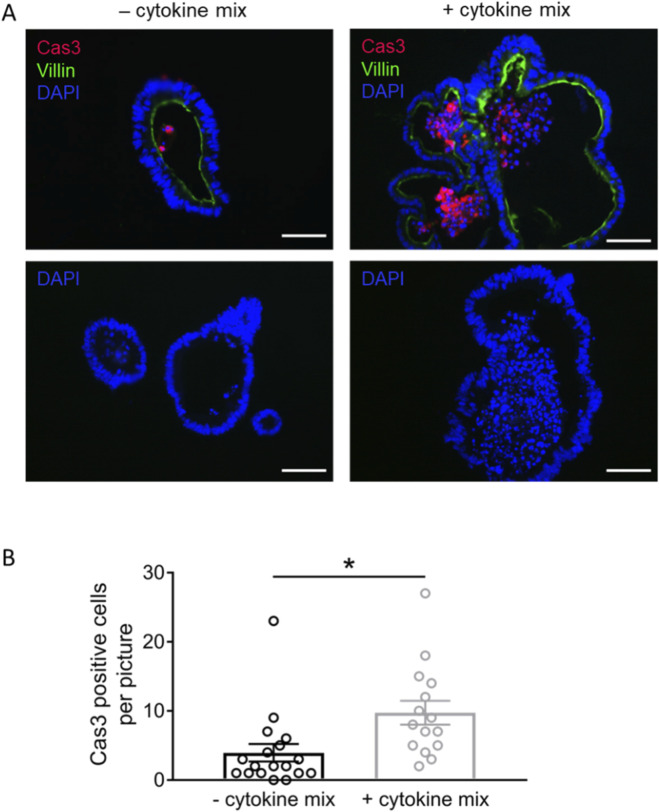
**(A)** Representative immunofluorescent stainings of cleaved caspase 3-positive cells (Cas3, combined with Cy3 donkey anti rabbit) in untreated organoids (- cytokine mix, left side) or organoids preincubated with the cytokine mix for 3 days (+ cytokine mix, right side). Villin (combined with Alexa488 donkey anti mouse) was used to distinguish the lumen. DAPI was used for nucleus staining. Negative controls without the respective primary antibody were performed in parallel (bottom row). Scale bar = 50 μm; pictures were taken with the Nikon 80i, 20x lens, NIS Elements 2.30 software (Nikon). **(B)** For blinded analysis, only cells positive for both Cas3 and DAPI were counted per image (n = 15–18 pictures under each experimental conditions) containing in total 24–37 organoids originating from three independent passages. Data are presented as mean (bars) ± SEM (lines) with single values as symbols. *p < 0.05 was set to be statistically significant.

TNFα is known to induce apoptosis via the type 1 receptor for this cytokine (TNFR1; Huyghe et al., 2023). To screen whether the number of apoptotic cells is changed in cytokine mix-treated organoids, immunofluorescent stainings against the activated form of caspase 3 (Cas3; Figure 6A), which represents a key enzyme in the apoptosis cascade (Lossi, 2022), was performed. The results showed that the number of Cas3-immunopositive cells in the organoid lumen increased by a factor of 2.5 after 3 days incubation with the cytokine mix (p < 0.05; Figure 6B).

### Changes in the expression of lipids involved in apoptosis

UHPLC MS/MS analyses were performed to investigate cytokine-induced alterations in lipid expression. Principal component analysis (PCA) revealed a clear segregation between treated and untreated intestinal organoids, indicating substantial changes in the lipidome and warranting further in-depth analyses. To explore these differences, hierarchical clustering was conducted, which confirmed that the lipid profiles of cytokine-treated organoids were statistically distinct from those of the untreated controls.

**FIGURE 7:**
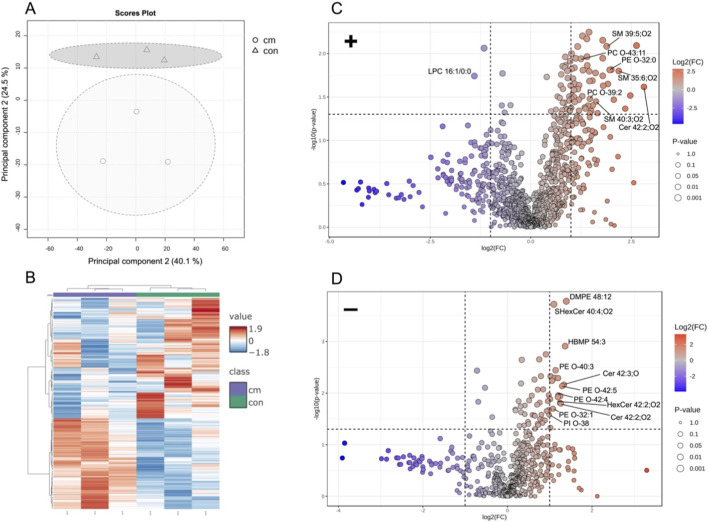
**(A)** Principal component analysis of the LC MS/MS data in positive-ion mode with three technical replicates reveals distinct separation between the control group and the treated samples. Percentage values represent the proportion of variance for each component. **(B)** Hierarchical clustering analysis between control group (con) and organoids treated with the cytokine mix (cm; exposure time 3 days) of the top 200 most differentially expressed lipids. **(C)** Volcano plot depicting the fold changes (log_2_ FC) in relation to significance (-Log_10_ *P*-value) of the lipids for the treated organoids (cm) relative to control (con) in positive-ion mode. A P-value threshold of p < 0.05 is shown. The upregulated lipids are displayed in red, the downregulated in blue and the lipids with no significant change in gray. **(D)** Volcano plot depicting the fold changes (log_2_ FC) in relation to significance (-log_10_ *P*-value) of the lipids for the treated organoids (cm) relative to control (con) in negative-ion mode. A P-value threshold of p < 0.05 is shown. The upregulated lipids are displayed in red, the downregulated in blue and the lipids with no significant change in gray.

Differential lipid expression was assessed using a Student’s t-test. Among the 1990 lipids detected in positive- and negative-ion modes, 58 were significantly altered following cytokine treatment (adjusted p < 0.05). Of these, 56 were upregulated and two downregulated (Supplementary Table S1). Notably, ether-linked lipids, sphingomyelins, and ceramides were markedly elevated in the treated group (Figure 7).

Consistent with our immunofluorescent observations, which showed increased apoptotic cells in the lumen of treated organoids, these findings suggest that cytokine exposure induces apoptosis. This is further supported by the elevated levels of sphingomyelins and ceramides, which are well-established mediators of apoptosis via the sphingomyelin signaling pathway (Haimovitz-Friedman et al., 1997a; Haimovitz-Friedman et al., 1997b).

### Ceramides were enriched in distinct regions of cytokine mix-treated organoids

**FIGURE 8:**
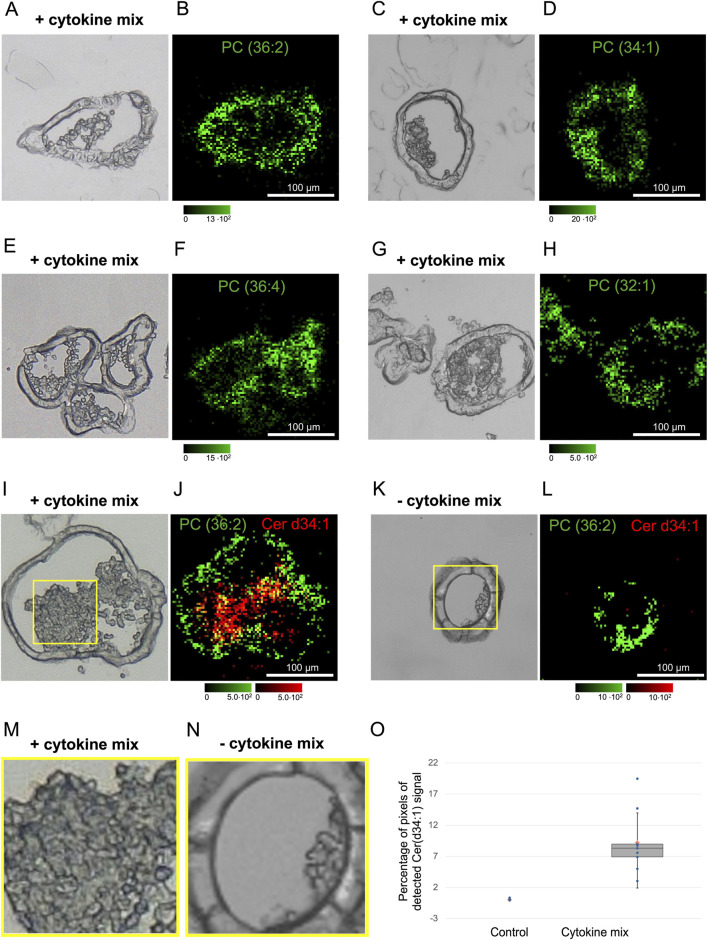
**(A)** Microscopic pictures of murine small intestinal organoids on the left **(A,C,E,G,I,K)** and corresponding MALDI MSI images on the right **(B, D, F, H, J,L)**. The lipids PC 36:2 ([M + Na]+, at m/z 808.5826), PC 34:1 ([M + H]+, at m/z 760.5845), PC 36:4 ([M + H]+, at m/z 782.5657) and PC 32:1 ([M + Na]+, at m/z 754.5363) are displayed in green. The microscopic picture **(I)** of a treated organoid (cytokine mix exposure for 3 days) shows a section (100 × 100 µm) of the lumen framed in yellow. An enlarged view (×2.6 magnification) of the section is displayed in **(M)**. The corresponding MALDI MSI image **(J)** displays Cer d34:1 ([M + H-H2O]+, at m/z 520.5087) in red and PC 36:1 ([M + H]+, at m/z 788.6149) in green. The image shows an accumulation of Cer d34:1 inside of the lumen. The Microscopic picture **(K)** of a control organoid shows a section (100 × 100 µm) of the lumen framed in yellow. An enlarged view (×2.6 magnification) of the section is displayed in **(N)**. The corresponding MALDI MSI image **(L)** displays Cer d34:1 ([M + H-H2O]+, at m/z 520.5055) in red and PC 36:2 ([M + H]+, at m/z 786.5991) in green. The lumen of the organoid is observed to be largely empty, exhibiting a significant absence of accumulation when compared to the treated sample. **(O)** Box plot with median (black line), mean (orange circle) and standard deviations (whiskers) of the number of pixels in which Cer d34:1 was detected, with a sample size of n = 9 organoids per group originating from three different passages.

AP-SMALDI MSI experiments were performed on cryosections of the organoid samples to determine whether the lipidomic differences detected by LC MS/MS between cytokine-treated and untreated organoids were associated with morphological changes at the molecular level. Two native color channels, red and green, were used to generate lossless overlay images from selected mass spectrometry signals. For illustration, Figures 8A–H shows the spatial distribution of different phosphatidylcholines (PCs) in green, predominantly localized within the epithelial layer and absent in the lumen of the organoids.

For several lipids identified by LC MS/MS, AP-SMALDI MSI revealed distribution patterns consistent with the optical images. Notably, ceramide (Cer) d34:1 was strongly enriched in cytokine-treated organoids compared with controls. As shown in Figure 8J, overlay images illustrate the spatial localization of Cer d34:1 in red, with accumulation specifically associated with regions containing dead cell material in the lumen of treated organoids (Figures 8I,M). In contrast, untreated organoids, which exhibited minimal luminal debris under light microscopy (Figures 8K,N), showed little to no Cer d34:1 signal (Figures 8L,O).

These findings were highly reproducible across both technical replicates (from the same organoid) and biological replicates (from independent organoids; Supplementary Figure S1). Together, these results demonstrate that cytokine treatment induces an accumulation of apoptotic cells within the organoid lumen and suggest that Cer d34:1 may serve as a potential molecular marker of cytokine-induced apoptosis.

## Discussion

### Selection of the cytokines used in the present study

Since proinflammatory cytokines play a crucial role in IBD (Neurath, 2014; Friedrich et al., 2019), we tested if a combination of TNFα, IL-1β and IFN-γ alters epithelial functions in murine intestinal organoids. Elevated levels of these cytokines (ranging from picomolar to nanomolar concentrations) were found in sera of IBD patients (Singh et al., 2016) as well as in colonic homogenates from mice with DSS-induced colitis (Bauer et al., 2010). When applied either as single cytokines (Pavlidis et al., 2022) or as a mix (e.g., TNFα, IL-1β and IFN-γ ranging from 10 to 30 ng/mL; Yokoi et al., 2024) to human colonic organoids, these cytokines induce changes in gene expression and transcriptome profiles similar to those observed in IBD patients. In the human intestinal cell line Caco-2, a mix of TNFα, IL-1β and IFN-γ has been shown to increase paracellular permeability in parallel to changes in mitogen-activated protein kinase (MAPK) signaling (Wang et al., 2008).

### Secretory changes of the organoids by cytokines

The mix of proinflammatory cytokines used in the present study (TNFα (100 ng/mL), IL-1β (20 ng/mL), IFN-γ (100 pg/mL)) induced pronounced changes in the morphology of the intestinal organoids. The organoids appeared to round off and their relative lumen size increased drastically (Figure 1A). Compared to the response of a single cytokine, i.e., when TNFα was applied alone (Pauer et al., 2024), the swelling was noticeably faster when a combination of proinflammatory cytokines was administered. The relative lumen size reached 50% of its final value after 4 h when organoids were treated with the cytokine mix (Figure 1B) and took up to 13 h in case of TNFα (Pauer et al., 2024). Synergistic interactions of cytokines during inflammation are well known (for review, see Gouwy et al., 2005). For example, in the human colonic epithelial cell line HT-29, the combination of IFN-γ with TNFα stimulates the secretion of the chemokine IP-10 (IFN-γ-inducible protein 10) more than 25x stronger than expected from the action of the individual cytokines when applied alone (Dwinell et al., 2001). In contrast, in isolated human distal colon, only additive interactions between TNFα and IL-1β on intestinal secretion were observed (Bode et al., 1998).

In contrast to data obtained for the prosecretory action of proinflammatory cytokines on human colon, where, e.g., the effect of TNFα is mediated by prostaglandins such as PGE_2_ (Schmitz et al., 1996), the swelling of the murine intestinal organoids was resistant against indometacin (Figure 2A), a blocker of cyclooxygenases, pointing to a direct effect of the cytokines at the epithelium. The most obvious explanation for these apparent conflicting results is the fact that prostaglandins are mostly produced within the submucosa (Craven and DeRubertis, 1986), e.g., by myofibroblasts (Beltinger et al., 1999), which are absent in the small intestinal organoids.

As it was already observed for TNFα (Pauer et al., 2024), the mechanism behind the cytokine mix-induced swelling of intestinal organoids involves the Na^+^-K^+^-2Cl^–^ cotransporter (NKCC) responsible for Cl^–^ uptake (Haas and Forbush, 2000; Keely and Barrett, 2022). Although there was only a slight upregulation of NKCC1 (Figure 2B), inhibition of this transporter with bumetanide clearly reduced the swelling induced by the cytokine mix (Figure 2A). However, due to the rapid onset (1 h) of the cytokine-induced swelling, it seems unlikely that transcriptional changes are responsible for the short-term secretory response.

### The cytokine mix alters Ca^2+^ signaling and Ca^2+^-dependent Cl^−^ secretion

Ca^2+^-dependent Cl^−^ secretion can be stimulated by acetylcholine or its stable derivative carbachol, where two phases of the increase in cytosolic Ca^2+^ levels can be distinguished: an initial release of Ca^2+^ from intracellular Ca^2+^ stores followed by an influx of Ca^2+^ from the extracellular space (Lindqvist et al., 1998). Carbachol activates muscarinic M_1_ and M_3_ receptors in the basolateral membrane which are coupled to G_q_ proteins activating a phosphatidylinositol 4,5-bisphosphate (PIP_2_)-specific phospholipase C (PLCβ3). The consequence is the cleavage of PIP_2_ into inositol-1,4,5-trisphosphate (IP_3_) and diacylglycerol. IP_3_ induces a release of Ca^2+^ from Ca^2+^-storing organelles such as the endoplasmic reticulum via IP_3_ receptors. The subsequent depletion of Ca^2+^ stores finally stimulates an influx of Ca^2+^ from the extracellular space via capacitative Ca^2+^ entry (Berridge, 1993). A central step in the activation of this capacitative Ca^2+^ entry is the interaction of the Ca^2+^ sensing protein Orai located in the cell membrane with STIM in so-called endoplasmic reticulum-plasma membrane junctions (Prakriya and Lewis, 2015). Gene expression of different molecules involved in Ca^2+^ signaling revealed an upregulation of IP_3_ receptor type 1 (IP_3_R1) and STIMs (reaching statistical significance only in case of STIM2) after 3 days cytokine exposure (Figure 5A). Interestingly, more upstream of Orai/STIM, central players of the cascade involved in the stimulation of Ca^2+^ release, i.e., the muscarinic receptor type M_3_, the α-subunit of G_q_ and PLCβ3 showed a significant downregulation (Figure 5B).

Functional experiments with the Ca^2+^-sensitive fluorescent dye, Fura-2, revealed time-dependent changes in the Ca^2+^ signaling after cytokine exposure when stimulated with carbachol. Short-term exposure (1 d) to the cytokine mix led to a drastic enhancement of the carbachol-induced increase in the Fura-2 signal, representing a rise in the cytosolic Ca^2+^ concentration (Figures 3A,B). The observed enhancement of extracellular Ca^2+^ influx faded (and lost statistical significance) after prolonged (≥3 days) incubation with the cytokines (Table 3). This might be explained - at least partially - by the observed downregulation of M_3_ receptor, G_q_α and PLCβ3 (Figure 5B). Hence, proinflammatory cytokines seem to enhance the cytosolic Ca^2+^ levels transiently over a short time period. This time dependency of the effect of proinflammatory cytokines might be an explanation for the conflicting results reported in the literature about changes in Ca^2+^ signaling under inflammatory conditions (see Introduction).

A different time dependency was observed when the experiments were performed in Ca^2+^-free buffer in which the Ca^2+^ influx from the extracellular space should not contribute to the remaining (smaller) increase in the Fura-2 ratio anymore. This response was transiently downregulated after 1 d cytokine mix incubation (and also after inhibition of nonselective cation channels involved in the capacitative Ca^2+^ entry in the intestinal epithelium (Frings et al., 1999) by La^3+^), but significantly upregulated in Ca^2+^-free buffer after prolonged exposure to the cytokine mix (≥3 days; Table 3). This fits well to the above-mentioned increase in gene expression of the IP_3_R1 and STIMs after 3 days incubation with the cytokine mix (Figure 5A). Taken together, these data clearly demonstrate the complexity of the changes in Ca^2+^ signaling cascades under inflammatory conditions.

### Apoptosis-linked changes in the lipidome induced by the cytokine mix

A well-known effect of cytokines, mainly TNFα, but also IL-1β or IFN-γ, is the induction of apoptosis (Woznicki et al., 2021; van Loo and Bertrand, 2023) contributing to the pathogenesis of IBD as indicated by the observation that the therapy with monoclonal antibodies against TNFα reduces epithelial apoptosis (Marini et al., 2003). Mechanistically, the first step in TNFα-induced apoptosis is the binding of the cytokine to the tumor necrosis factor receptor type 1 (TNFR1) containing a “death-domain”. The consequence is the formation of a membrane-bound array of proteins, the so-called complex I, which activates proinflammatory signaling pathways such as the nuclear factor κB pathway. Subsequently, a cytoplasmic array of proteins, the complex II, is formed. The following activation of caspases (cysteinyl-aspartate specific proteases) leads to the cleavage of cellular proteins and fragmentation of the cell nucleus (van Loo and Bertrand, 2023). Activation of caspase 3 is a point of no return in the apoptosis cascade and, indeed, the number of caspase 3-immunopositive cells was significantly enhanced in intestinal organoids pre-treated with the cytokine mix (Figure 6B).

Beside changes in cellular protein patterns or nucleic acids, also modifications of the lipid composition of the intestinal epithelium and/or the intestinal mucus have been found under inflammatory conditions such as an increase in ceramide levels or alterations in the ratio of phosphatidylcholine/phosphatidylethanolamine (for review see Boldyreva et al., 2021). Furthermore, oral treatment with phospholipids with the goal to improve the protective mucus layer of the epithelium proved to be beneficial in patients with ulcerative colitis (Stremmel et al., 2005). Therefore, we performed an untargeted analysis of the epithelial lipidome using LC MS/MS followed by MALDI MSI to localize changes in lipid distribution induced by cytokine treatment compared with untreated control organoids. Liquid chromatography coupled with tandem mass spectrometry (LC MS/MS) enables a comprehensive, untargeted profiling of lipids present in organoid samples without prior knowledge of their composition. Moreover, MS/MS fragmentation provides detailed structural information, allowing accurate identification of lipid species.

Among the 1990 lipids analyzed by LC MS/MS, 58 were differentially expressed following cytokine treatment, with 56 upregulated and two downregulated (Figure 7). Within the upregulated cohort, three major lipid groups could be distinguished. The first group comprised ether-linked phospholipids, which are typically enriched in lipid rafts. Ether-linked phospholipids tend to reduce membrane fluidity due to their tighter packing compared to ester-linked phospholipids and are involved in cell signaling and differentiation (Dean and Lodhi, 2018). It is also known that ether-linked glycerophospholipids are increased during apoptosis (Fuchs et al., 2007), which fits well to the upregulation of ether-linked lipids in intestinal organoids after cytokine mix incubation (Figure 7; Supplementary Figure S2). The second group consisted of sphingomyelins (N-acyl-sphingosine-1-phosphorylcholine), which are predominantly localized in plasma membranes (Haimovitz-Friedman et al., 1997a). The third group included ceramides, generated through the cleavage of membrane sphingomyelin by sphingomyelinases (a class of phospholipase C enzymes; Haimovitz-Friedman et al., 1997b). The increased production of ceramides induced by proinflammatory cytokines such as TNFα has been reported previously (Obeid et al., 1993). As intracellular second messengers, ceramides play essential roles in regulating cell proliferation, differentiation, and apoptosis (Haimovitz-Friedman et al., 1997b).

At first glance, the simultaneous upregulation of both ceramides and their precursor sphingomyelins might seem counterintuitive. However, it has been shown that TNFα also stimulates *de novo* ceramide synthesis by upregulating ceramide synthases, which catalyze the N-acylation of sphingosine (Hernández-Corbacho et al., 2015). Therefore, the observed ceramide accumulation in the lipidome likely results from a combined effect of increased sphingomyelin turnover and enhanced *de novo* synthesis.

To distinguish viable epithelial cells from apoptotic debris within the organoid lumen, MALDI MSI was applied to visualize the distribution of phosphatidylcholines (PCs), major structural components of cell membranes (Figure 8). Based on these observations, we further investigated the spatial distribution of Cer d34:1, one of the ceramides identified via LC MS/MS. Strikingly, Cer d34:1 was strongly enriched within luminal debris derived from cytokine-induced apoptosis of epithelial cells, whereas it was rarely detectable in untreated control organoids (Figures 8J,L,O). These findings suggest that Cer d34:1 may serve as a potential molecular marker for cytokine-induced apoptotic cells.

Taken together, modelling intestinal inflammation with cytokine mix-treated intestinal organoids fits well to the apoptosis cascade reported in the literature.

### Contradictory findings in epithelial barrier after cytokine mix

Interestingly, the barrier function of the epithelium, at least when regarding the expression of different claudins, was not impaired. These constitutive components of the tight junctions can be divided into barrier- and pore-forming claudins (Günzel and Yu, 2013). Inflammatory bowel diseases are usually accompanied by a decreased barrier function either by reduced expression of tightening barrier proteins, such as claudin-3 or claudin-4, or by enhanced expression of pore-forming proteins like claudin-2 (Barmeyer et al., 2017). Also, proinflammatory cytokines such as TNFα and IFN-γ are well-known to enhance epithelial barrier permeability in different intestinal cell line and animal models. These cytokines lead via receptor-dependent activation of NFκB/ERK/p38 pathways and enhanced myosin light chain kinase expression and activity to tight junction remodeling and endocytosis of occludin and zonula occludens 1 (ZO-1), two other important proteins for the leak pathway of epithelial barrier (Horowitz et al., 2023; Meyer et al., 2023). However, in the isolated organoids, there was no hint for any alterations in gene expression of claudins, belonging to the pore pathway (Horowitz et al., 2023), correlated with an increased permeability of the epithelial barrier. The only statistically significant changes induced by the cytokines consisted in the upregulation of claudin-3 and claudin-4 (Figure 5C) implicating a tighter epithelial barrier. Speculatively, this might represent a counter-regulation of the organoid’s epithelium in order to withstand the increased luminal pressure after the massive swelling, but it clearly does not mirror the *in vivo* situation, which is characterized by a “leaky gut” phenomenon in IBD (Sandle and Rajendran, 2025). Another explanation for this discrepancy might be that non-epithelial cells, e.g., immune cells, are missing in this model since it is known that released immune cell mediators such as proteases enhance the epithelial permeability, e.g., by disrupting ZO-1 (for a review, see van Spaendonk et al., 2017). Furthermore, it has to be considered that cytokines and other inflammatory mediators do not only drive intestinal inflammation, e.g., by impairment of the epithelial barrier, but they are also involved in proliferation and tissue repair (Crifo and MacNaughton, 2022).

### Limitations of the current study and outlook

Albeit the current study shows how a mix of TNFα, IL-1β and IFN-γ induces secretion and apoptosis and changes intracellular Ca^2+^ in a time-dependent manner, there are some limitations, e.g., in study design. Due to the numerous data from other published literature, only defined cytokine concentrations and only three different time points were chosen for experiments. The effect of other pro- and anti-inflammatory cytokines, which are involved in IBD, would be interesting to investigate, but this was out of the scope of the current study. Nevertheless, intestinal organoids are a useful model to study, e.g., long-term effects of cytokines, which is not possible when using primary cultures such as isolated intestinal crypts. Furthermore, the use of organoids can reduce the number and suffering of animals according to the 3R principle (Russell and Burch, 1960) instead of classical models of chemically induced colitis (Katsandegwaza et al., 2022). However, they have also limitations, mainly the missing interaction of the epithelium with the microbiome and neighboring cell types. Cell types with important interactions during inflammation are not only immune cells such as macrophages, lymphocytes or mast cells, which are found in the gut wall under physiologic conditions (Cader and Kaser, 2013), but also enteric neurons, connective tissue or smooth muscle cells. For example, myofibroblast produce eicosanoids such as PGE_2_ (Shao et al., 2006) or secretomotor neurons of the enteric nervous system release neurotransmitters leading to a spontaneous Cl^−^ secretion (Andres et al., 1985). Thus, organoids can be an important tool to study, e.g., regulation of epithelial signaling cascades or molecules, but there is clearly the need to build up more complex organoid coculture systems or organ-on-a-chip system (Sandle and Rajendran, 2025) to investigate complex intercellular communication pathways under inflammatory conditions.

## Data Availability

The raw data supporting the conclusions of this article will be made available by the authors, without undue reservation.
