# Supplementary material for: Proinflammatory cytokines modify Ca2+ signaling and induce an apoptotic lipidome in murine small intestinal organoids
Source: Front Physiol. 2026 Feb 6;17:1738447. doi: 10.3389/fphys.2026.1738447 (PMC12920202; doi:10.3389/fphys.2026.1738447)
Supplement: Supplementary file 1 [file DataSheet1.docx]

Supporting information

Proinflammatory cytokines modify Ca^2+^ signaling and induce an apoptotic lipidome in murine small intestinal organoids

Svenja Mareike Pauer^1^, Alina Schindler^2^, Parviz Ghezellou^2,3,4^, Bernhard Spengler^2^, Martin Diener^1^, Jasmin Ballout^1*^

^1^ Institute for Veterinary Physiology and Biochemistry, Justus Liebig University Giessen, Giessen, Germany

^2^ Institute for Inorganic and Analytical Chemistry, Justus Liebig University Giessen, Giessen, Germany

^3^ Institute of Molecular Systems Medicine, Goethe University, Frankfurt am Main, Germany

^4^ Institute for Cardiovascular Physiology, Goethe University, Frankfurt am Main, Germany

# **Supplementary Protocol 1**: Instrumentation parameters for AP-SMALDI MSI experiments.

AP-SMALDI MSI measurements were performed in 2D pixel mode with 50 laser pulses per pixel at 343 nm wavelength and a pulse rate of 100 Hz. For 3 µm step size experiments, the mass range was set to *m*/*z* 350-1200 with a mass resolution of 240,000 at *m*/*z* 200. The mass spectrometer was operated in positive-ion mode, and an internal lock-mass calibration was performed by using the ion signal of the DHB matrix cluster at *m*/*z* 716.1245 ([5DHB+NH_4_-4H_2_O]). The capillary temperature was set to 250 °C and the ion injection time to 500 ms. S-lense level was set to 80 arbitraty units and the acceleration voltage to ±3 kV.

# **Supplementary Table 1**: Up- and downregulated lipids in cytokine-treated samples in comparison to control samples detected via UHPLC MS/MS. Upregulated lipids include ceramides (Cer), dimethylphosphatidylethanolamines (DMPE), hexosylceramides (HexCer), hemibismonoacylglycerophosphates (HBMP), phosphatidylcholines (PC), ether-linked phosphatidylcholines (PC O), phosphatidylethanolamines (PE), ether-linked phosphatidylethanolamines (PE O), 1Z-alkenyl ether-linked phosphatidylethanolamines (PE P), ether-linked phosphatidylinositol (PI O), sulfated hexosylceramides (SHexCer), sphingomyelin (SM), sterols (ST), and triglycerides (TG). Downregulated lipids include HexCer and lysophosphatidylcholine (LPC).

| Upregulated | Downregulated |
| --- | --- |
| TG 18:0/18:1/18:1 | LPC 16:1/0:0 |
| SM 39:5;O2 | HexCer 18:0;O2/16:0 |
| ST 29:2;O |  |
| TG 14:0_16:0/16:1 |  |
| PC O-43:11 |  |
| PC 22:1/20:4 |  |
| PC O-18:1/18:2 |  |
| PC O-20:0/20:4 |  |
| PE O-32:0 |  |
| TG 14:0/15:0/16:1 |  |
| TG 15:0/16:0/18:1 |  |
| PE P-16:0/18:3 |  |
| SM 35:6;O2 |  |
| Cer 18:1;O2/24:1 |  |
| SM 40:7;O2 |  |
| PE P-18:1/16:1 |  |
| TG 16:0/15:1/16:1 |  |
| PC O-20:1/20:3 |  |
| TG 14:0/16:0/18:1 |  |
| SM 38:3;O2 |  |
| TG 14:1/16:1/18:1 |  |
| PE 36:3 |  |
| PC 20:3/22:3 |  |
| PE 18:1/18:2 |  |
| SM 40:3;O2 |  |
| DMPE 24:6/24:6 |  |
| SHexCer 40:4;O2 |  |
| HBMP 18:1/18:1/18:1 |  |
| PE O-18:0/22:3 |  |
| DMPE 22:0/18:1 |  |
| DMPE 14:0/16:0 |  |
| HBMP 16:1/18:1/18:1 |  |
| PE 20:4/28:0;O |  |
| Cer 18:2;O2/24:1 |  |
| PE O-22:1/20:4 |  |
| PE O-18:1/14:0 |  |
| PI O-18:0/20:3 |  |
| PE O-22:1/20:3 |  |
| PE 20:4/24:0;O |  |
| Cer 18:1;O2/24:1 |  |
| HexCer 42:2;O2 |  |
| RIKEN P-VS1 ID-10479 from Mouse_Plasma_WT_N_Ctr |  |
| RIKEN P-VS1 ID-18069 from Mouse_Macrophage_WT_N_F1 |  |
| RIKEN P-VS1 ID-5931 from Mouse_Macrophage_WT_N_F1AA |  |
| RIKEN P-VS1 ID-6693 from Mouse_Feces_WT_N_Ctr |  |
| RIKEN P-VS1 ID-14241 from Mouse_Kidney_WT_N_F1AA |  |
| RIKEN P-VS1 ID-4949 from Mouse_Muscle_WT_CTX0_Ctr |  |
| RIKEN P-VS1 ID-18323 from Mouse_Feces_WT_N_Ctr |  |
| RIKEN P-VS1 ID-16048 from Mouse_Macrophage_WT_N_F1DHA |  |
| RIKEN P-VS1 ID-7318 from Mouse_AdrenalGlands_WT_N_Ctr |  |
| RIKEN P-VS1 ID-18373 from Cell_HEK293_WT_N_Ctr |  |
| RIKEN P-VS1 ID-12952 from Mouse_Feces_WT_N_Ctr |  |
| RIKEN P-VS1 ID-6396 from Cell_C2C12_WT_N_12hCtr |  |
| RIKEN P-VS1 ID-8933 from Mouse_Feces_WT_N_Ctr |  |
| RIKEN P-VS1 ID-7761 from Mouse_Feces_WT_N_Ctr |  |
| RIKEN N-VS1 ID-6927 from Mouse_Brain_WT_N_F1AA |  |

# **Supplementary Table 2**: MS and MS/MS parameters (in brackets) used for identification of lipids after C18-separation.

| Parameter | Setting |
| --- | --- |
| Mass resolution | 120,000 (45,000) |
| AGC target | 1e6 (1e5) |
| Max. injection time | 75 ms (75 ms) |
| Scan range | *m*/*z* 350 – 1200 (200-1200) |
| Top N | 10 |
| Isolation window | *m*/*z* 1 |
| Stepped NCE | 20; 25; 30 |
| Dynamic exclusion | 6 s |
| Spray voltage | ±3.5 kV |
| Capillary temperature | 300 |
| S-lense setting | 80 |

# **Supplementary Table 3**: MS Dial parameter settings for data anylsis of the obtained UHPLC MS/MS data.

| Category | Parameter | Setting |
| --- | --- | --- |
| Data collection | MS tolerance | 0.01 Da |
|  | MS/MS tolerance | 0.025 Da |
|  | Retention time begin | 0 min |
|  | Retention time end | 38 min |
|  | MS mass range begin | 350 |
|  | MS mass range end | 1200 |
|  | MS/MS mass range begin | 0 |
|  | MS/MS mass range end | 1200 |
| Peak detection | Minimum peak height | 10,000 amplitude |
|  | Mass slice width | 0.4 Da |
|  | Smoothing method | Linear weighted moving average |
|  | Smoothing level | 8 scans |
|  | Minimum peak width | 5 scans |
| Deconvolution | Sigma window value | 0.5 |
|  | MS/MS abundance cut off | 0 amplitude |
|  | Exclude after precurser ion | Check |
|  | Keep the isotopic ions until | 1 |
| Identification | Retention time tolerance | 100 min |
|  | Accurate-mass tolerance (MS) | 0.01 Da |
|  | Accurate mass tolerance (MS/MS) | 0.025 Da |
|  | Identification score cut off | 80% |
|  | Use retention time for scoring | Check |
|  | Use retention time for filtering | Check |
| Alignment | Reference file | Quality control |
|  | Retention time tolerance | 2 min |
|  | MS tolerance | 0.01 |
|  | Retention time factor | 0.5 |
|  | MS factor | 0.5 |
|  | Peak count filter | 0% |
|  | %N detected in at least one group | 60% |
|  | Remove features based on blank information | Check |
|  | Sample max / blank average | 5-fold change |


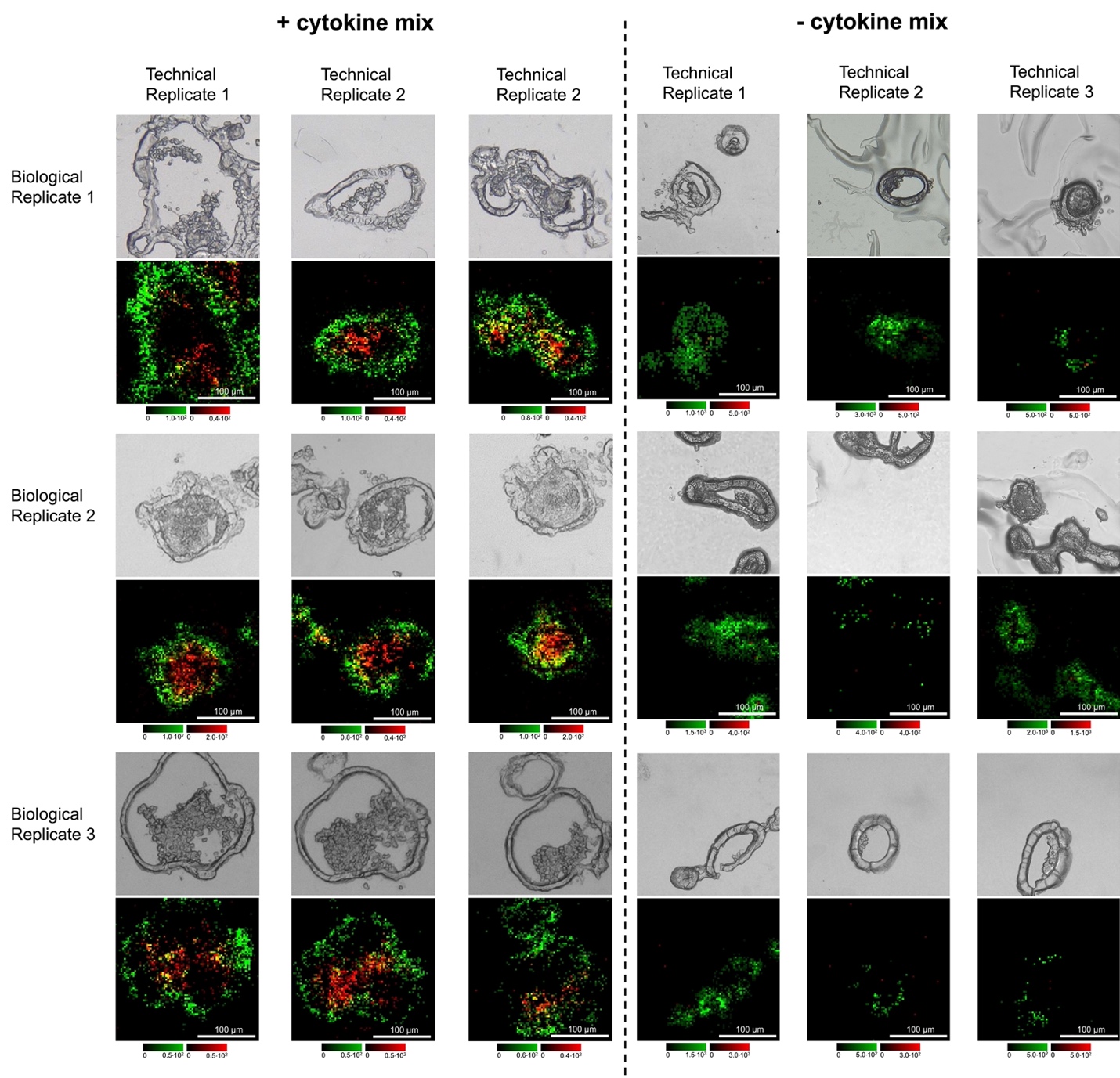


# **Supplementary Figure 1**: Microscopic pictures of three biological (defined as independent passages) and three technical (defined as different organoids from the same passage) replicates at the top rows (gray). The organoids shown on the left are treated with the cytokine mix and the organoids shown on the right are untreated control samples. Corresponding AP-SMALDI MSI images are shown below. PC 36:2 ([M+Na]^+^, at *m/z* 808.5827) is displayed in green and Cer d34:1 ([M+H-H_2_O]^+^, at *m/z* 520.5088) is displayed in red.


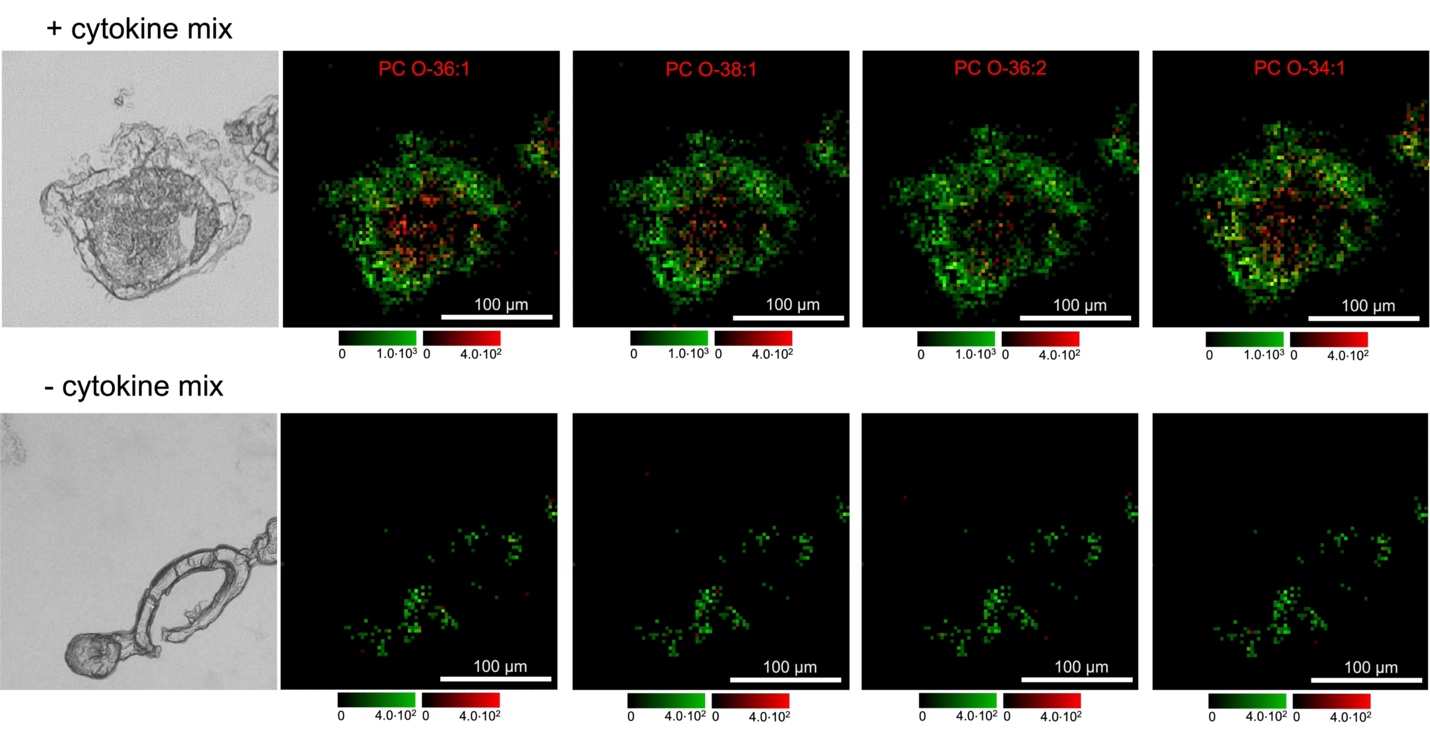


# **Supplementary Figure 2:** Microscopic pictures of a treated organoid (top row) and an untreated organoid (bottom row). The corresponding AP-SMALDI MSI images are shown on the right. PC 36:2 ([M+Na]+, at m/z 808.5827) is displayed in green and a variety of ether-linked PC´s are shown in red: PC O-36:1 ([M+H]+, at m/z 774.6358), PC O-38:1 ([M+H]+, at m/z 802.6671), PC O-36:2 ([M+H]+, at m/z 772.6215) and PC O-34:1 ([M+Na]+, at m/z 768.5878).
